# Tris(1,2-dimeth­oxy­ethane-κ^2^ *O*,*O*′)iodidocalcium iodide

**DOI:** 10.1107/S160053681200075X

**Published:** 2012-01-18

**Authors:** Siou-Wei Ou, Wei-Yi Lu, Hsuan-Ying Chen

**Affiliations:** a Department of Medicinal and Applied Chemistry, Kaohsiung Medical University, Kaohsiung 807, Taiwan; b Department of Chemistry, National Chung Hsing University, Taichung 402, Taiwan

## Abstract

In the title complex, [CaI(C_4_H_10_O_2_)_3_]I, the Ca^II^ atom is seven-coordinated by six O atoms from three 1,2-dimeth­oxy­ethane (DME) ligands and one iodide anion in a distorted penta­gonal–bipyramidal geometry. The I atom and one of the O atoms from a DME ligand lie in the axial positions while the other O atoms lie in the basal plane. The other iodide anion is outside the complex cation.

## Related literature

For background to polylactide and its copolymers, see: Ha & Gardella (2005 ▶); Simpson *et al.* (2008 ▶). For ring-opening polymerization of lactides with calcium-based catalysts, see: Chen *et al.* (2007 ▶); Chisholm *et al.* (2003 ▶, 2004 ▶); Darensbourg *et al.* (2002 ▶, 2003*a* ▶,*b* ▶); Zhong *et al.* (2001 ▶).
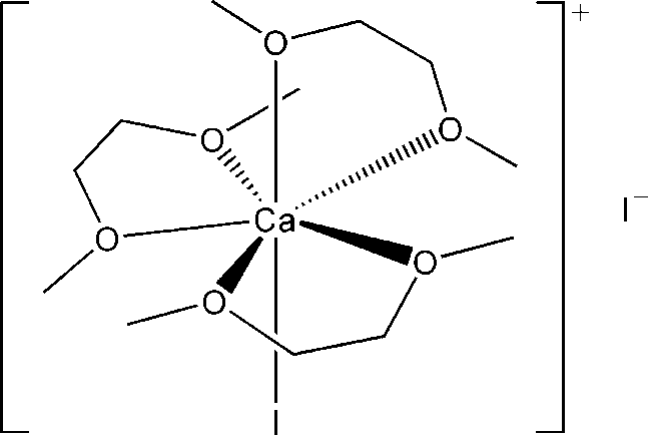


## Experimental

### 

#### Crystal data

[CaI(C_4_H_10_O_2_)_3_]I
*M* *_r_* = 564.24
Monoclinic, 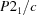

*a* = 12.1503 (6) Å
*b* = 10.7767 (5) Å
*c* = 16.2295 (8) Å
β = 99.514 (1)°
*V* = 2095.86 (18) Å^3^
*Z* = 4
Mo *K*α radiation
μ = 3.26 mm^−1^
*T* = 100 K
0.23 × 0.23 × 0.15 mm

#### Data collection

Bruker APEXII CCD diffractometer
Absorption correction: multi-scan (*SADABS*; Bruker, 2001 ▶) *T*_min_ = 0.522, *T*_max_ = 0.647
14842 measured reflections
4791 independent reflections
4627 reflections with *I* > 2σ(*I*)
*R*_int_ = 0.017

#### Refinement

*R*[*F* ^2^ > 2σ(*F* ^2^)] = 0.016
*wR*(*F* ^2^) = 0.043
*S* = 1.19
4791 reflections
196 parameters
H-atom parameters constrained
Δρ_max_ = 0.35 e Å^−3^
Δρ_min_ = −0.91 e Å^−3^

### 

Data collection: *APEX2* (Bruker, 2007 ▶); cell refinement: *SAINT* (Bruker, 2007 ▶); data reduction: *SAINT*; program(s) used to solve structure: *SHELXTL* (Sheldrick, 2008 ▶); program(s) used to refine structure: *SHELXTL*; molecular graphics: *SHELXTL*; software used to prepare material for publication: *SHELXTL*.

## Supplementary Material

Crystal structure: contains datablock(s) global, I. DOI: 10.1107/S160053681200075X/hy2505sup1.cif

Structure factors: contains datablock(s) I. DOI: 10.1107/S160053681200075X/hy2505Isup2.hkl

Additional supplementary materials:  crystallographic information; 3D view; checkCIF report

## Figures and Tables

**Table 1:** Selected bond lengths (Å)

Ca1—O1	2.4046 (12)
Ca1—O2	2.3872 (12)
Ca1—O3	2.4871 (11)
Ca1—O4	2.4415 (11)
Ca1—O5	2.4254 (12)
Ca1—O6	2.5138 (11)
Ca1—I1	3.0525 (3)

**Fig. 1.**
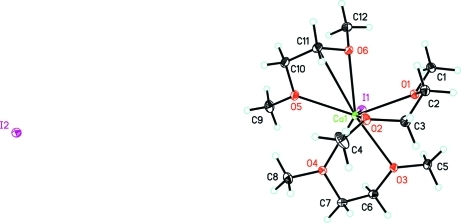
The molecular structure of the title compound. Displacement ellipsoids are shown at the 40% probability level.

## supplementary crystallographic information

### Comment

Polylactide and its copolymers are so popular and have been researched widely because of their diversiform applications in biomaterial fields (Ha & Gardella, 2005; Simpson *et al.*, 2008). The ring-opening polymerization (ROP) is the major way to polymerize lactides. Because of the biomaterial purpose the catalysts with non-cytotoxic character are required and calcium is paid much attention for this reason. Recently, ROP of lactides with calcium-based catalysts has been reported (Chen *et al.*, 2007; Chisholm *et al.*, 2003, 2004; Darensbourg *et al.*, 2002, 2003*a*,b; Zhong *et al.*, 2001) and calcium bis[bis(trimethylsilyl)amide] or calcium iodide is the common precursor. But the low dissolution of calcium iodide in organic solvent is its problem. In this paper we used 1,2-dimethoxyethane (DME) as ligand and solvent to react with calcium iodide, and the solution was refluxed until it showed clear. When the temperature of the solution was cooled to 0°C, the rectangular crystals, [Ca(DME)_3_I]I, appeared. The compound was dissoluble in DME, THF and CH_2_Cl_2_. The low dissolution problem of calcium iodide in organic solvent was solved through the synthesis of [Ca(DME)_3_I]I, if calcium iodide was the necessary precursor.

In the title compound, the Ca^II^ atom is heptacoordinated with a distorted pentagonal-bipyramidal geometry, in which five O atoms occupied the basal positions are almost coplanar . One Cl atom and one O atom lie on the axial positions. The Ca—O distances are 2.3872 (12)–2.5138 (11) Å and the Ca—I distance is 3.0525 (3) Å (Table 1).

### Experimental

A suspension of calcium iodide (2.94 g, 10 mmol) in 1,2-dimethoxyethane (10 ml) was stirred and refluxed until it showed clear. When the solution was cooled down to 0°C slowly, the rectangular colorless crystals appeared. The solution was filtered to remove the excess 1,2-dimethoxyethane. Volatile materials were then removed under a vacuum. Yield: 78%.

### Refinement

H atoms were positioned geometrically and refined as riding atoms, with C—H = 0.99 (CH_2_) and 0.98 (CH_3_) Å and with *U*_iso_(H) = 1.2(1.5 for methyl)*U*_eq_(C).

### Crystal data

**Table:**

[CaI(C_4_H_10_O_2_)_3_]I	*F*(000) = 1104
*M**_r_* = 564.24	*D*_x_ = 1.788 Mg m^−^^3^
Monoclinic, *P*2_1_/*c*	Mo *K*α radiation, λ = 0.71073 Å
Hall symbol: -P 2ybc	Cell parameters from 9874 reflections
*a* = 12.1503 (6) Å	θ = 2.0–28.0°
*b* = 10.7767 (5) Å	µ = 3.26 mm^−^^1^
*c* = 16.2295 (8) Å	*T* = 100 K
β = 99.514 (1)°	Blocks, colourless
*V* = 2095.86 (18) Å^3^	0.23 × 0.23 × 0.15 mm
*Z* = 4	

### Data collection

**Table:**

Bruker APEXII CCD diffractometer	4791 independent reflections
Radiation source: fine-focus sealed tube	4627 reflections with *I* > 2σ(*I*)
graphite	*R*_int_ = 0.017
φ and ω scans	θ_max_ = 27.5°, θ_min_ = 1.7°
Absorption correction: multi-scan (*SADABS*; Bruker, 2001)	*h* = −15→15
*T*_min_ = 0.522, *T*_max_ = 0.647	*k* = −13→14
14842 measured reflections	*l* = −21→20

### Refinement

**Table:**

Refinement on *F*^2^	Primary atom site location: structure-invariant direct methods
Least-squares matrix: full	Secondary atom site location: difference Fourier map
*R*[*F*^2^ > 2σ(*F*^2^)] = 0.016	Hydrogen site location: inferred from neighbouring sites
*wR*(*F*^2^) = 0.043	H-atom parameters constrained
*S* = 1.19	*w* = 1/[σ^2^(*F*_o_^2^) + (0.0191*P*)^2^ + 0.9088*P*] where *P* = (*F*_o_^2^ + 2*F*_c_^2^)/3
4791 reflections	(Δ/σ)_max_ = 0.005
196 parameters	Δρ_max_ = 0.35 e Å^−^^3^
0 restraints	Δρ_min_ = −0.91 e Å^−^^3^

### Special details

**Table:**

Geometry. All e.s.d.'s (except the e.s.d. in the dihedral angle between two l.s. planes) are estimated using the full covariance matrix. The cell e.s.d.'s are taken into account individually in the estimation of e.s.d.'s in distances, angles and torsion angles; correlations between e.s.d.'s in cell parameters are only used when they are defined by crystal symmetry. An approximate (isotropic) treatment of cell e.s.d.'s is used for estimating e.s.d.'s involving l.s. planes.
Refinement. Refinement of *F*^2^ against ALL reflections. The weighted *R*-factor *wR* and goodness of fit *S* are based on *F*^2^, conventional *R*-factors *R* are based on *F*, with *F* set to zero for negative *F*^2^. The threshold expression of *F*^2^ > σ(*F*^2^) is used only for calculating *R*-factors(gt) *etc*. and is not relevant to the choice of reflections for refinement. *R*-factors based on *F*^2^ are statistically about twice as large as those based on *F*, and *R*- factors based on ALL data will be even larger.

### Fractional atomic coordinates and isotropic or equivalent isotropic displacement parameters (Å^2^)

**Table:**

	*x*	*y*	*z*	*U*_iso_*/*U*_eq_	
Ca1	0.74096 (2)	0.25280 (3)	0.987454 (18)	0.01103 (6)	
I1	0.589004 (9)	0.206685 (9)	1.116467 (6)	0.01643 (4)	
I2	0.169983 (9)	0.297669 (9)	0.264013 (7)	0.01675 (4)	
O1	0.91500 (9)	0.19272 (10)	1.07103 (7)	0.0141 (2)	
O2	0.89431 (10)	0.28746 (10)	0.91541 (7)	0.0165 (2)	
O3	0.80225 (9)	0.44050 (10)	1.07187 (7)	0.0152 (2)	
O4	0.66028 (9)	0.44389 (10)	0.92287 (7)	0.0163 (2)	
O5	0.60542 (9)	0.17389 (11)	0.87284 (7)	0.0164 (2)	
O6	0.77110 (9)	0.02920 (10)	0.95308 (7)	0.0155 (2)	
C1	0.92225 (14)	0.09314 (15)	1.13105 (10)	0.0178 (3)	
H1A	0.8532	0.0897	1.1548	0.027*	
H1B	0.9857	0.1078	1.1758	0.027*	
H1C	0.9328	0.0143	1.1033	0.027*	
C2	1.01205 (14)	0.19625 (14)	1.03155 (10)	0.0166 (3)	
H2A	1.0198	0.1169	1.0024	0.020*	
H2B	1.0797	0.2086	1.0740	0.020*	
C3	0.99948 (14)	0.30183 (15)	0.96992 (11)	0.0185 (3)	
H3A	1.0016	0.3821	0.9998	0.022*	
H3B	1.0613	0.3005	0.9371	0.022*	
C4	0.88796 (16)	0.3613 (2)	0.84086 (12)	0.0305 (4)	
H4A	0.8171	0.3450	0.8040	0.046*	
H4B	0.9500	0.3397	0.8119	0.046*	
H4C	0.8925	0.4494	0.8559	0.046*	
C5	0.84956 (14)	0.42976 (16)	1.15957 (10)	0.0193 (3)	
H5A	0.9184	0.3808	1.1655	0.029*	
H5B	0.7960	0.3885	1.1894	0.029*	
H5C	0.8663	0.5127	1.1831	0.029*	
C6	0.71418 (14)	0.53093 (15)	1.05910 (10)	0.0187 (3)	
H6A	0.7380	0.6076	1.0907	0.022*	
H6B	0.6471	0.4981	1.0790	0.022*	
C7	0.68799 (15)	0.55864 (15)	0.96726 (11)	0.0200 (3)	
H7A	0.6244	0.6170	0.9559	0.024*	
H7B	0.7534	0.5975	0.9484	0.024*	
C8	0.61548 (15)	0.46988 (16)	0.83621 (10)	0.0213 (3)	
H8A	0.6066	0.3920	0.8046	0.032*	
H8B	0.6668	0.5246	0.8127	0.032*	
H8C	0.5427	0.5106	0.8327	0.032*	
C9	0.48713 (14)	0.20029 (17)	0.85691 (12)	0.0231 (4)	
H9A	0.4718	0.2731	0.8893	0.035*	
H9B	0.4460	0.1287	0.8733	0.035*	
H9C	0.4635	0.2169	0.7972	0.035*	
C10	0.63075 (14)	0.06361 (15)	0.82961 (10)	0.0178 (3)	
H10A	0.6216	0.0796	0.7688	0.021*	
H10B	0.5797	−0.0045	0.8393	0.021*	
C11	0.74998 (14)	0.02824 (15)	0.86297 (10)	0.0179 (3)	
H11A	0.7652	−0.0557	0.8427	0.021*	
H11B	0.8010	0.0873	0.8417	0.021*	
C12	0.71396 (15)	−0.07020 (15)	0.98863 (11)	0.0201 (3)	
H12A	0.7403	−0.0744	1.0490	0.030*	
H12B	0.7294	−0.1491	0.9628	0.030*	
H12C	0.6334	−0.0544	0.9782	0.030*	

### Atomic displacement parameters (Å^2^)

**Table:**

	*U*^11^	*U*^22^	*U*^33^	*U*^12^	*U*^13^	*U*^23^
Ca1	0.01102 (14)	0.01151 (14)	0.01052 (13)	−0.00026 (10)	0.00169 (10)	−0.00006 (10)
I1	0.01616 (6)	0.01888 (6)	0.01554 (6)	−0.00274 (3)	0.00641 (4)	0.00003 (3)
I2	0.01794 (6)	0.01398 (6)	0.01738 (6)	−0.00181 (3)	0.00008 (4)	0.00072 (3)
O1	0.0140 (5)	0.0152 (5)	0.0132 (5)	0.0014 (4)	0.0025 (4)	0.0007 (4)
O2	0.0161 (6)	0.0204 (6)	0.0132 (5)	−0.0004 (4)	0.0032 (4)	0.0048 (4)
O3	0.0160 (5)	0.0152 (5)	0.0132 (5)	0.0025 (4)	−0.0010 (4)	−0.0025 (4)
O4	0.0204 (6)	0.0127 (5)	0.0144 (5)	0.0004 (4)	−0.0013 (4)	0.0010 (4)
O5	0.0132 (5)	0.0182 (5)	0.0167 (5)	−0.0002 (4)	−0.0009 (4)	−0.0025 (4)
O6	0.0169 (5)	0.0137 (5)	0.0148 (5)	−0.0023 (4)	−0.0003 (4)	−0.0011 (4)
C1	0.0223 (8)	0.0171 (7)	0.0135 (7)	0.0011 (6)	0.0011 (6)	0.0029 (6)
C2	0.0134 (7)	0.0203 (8)	0.0165 (8)	0.0019 (6)	0.0034 (6)	−0.0010 (6)
C3	0.0142 (8)	0.0198 (8)	0.0216 (8)	−0.0027 (6)	0.0032 (6)	0.0001 (6)
C4	0.0237 (9)	0.0439 (12)	0.0260 (9)	0.0068 (8)	0.0102 (7)	0.0214 (8)
C5	0.0234 (8)	0.0192 (8)	0.0135 (7)	0.0005 (6)	−0.0021 (6)	−0.0037 (6)
C6	0.0186 (8)	0.0149 (7)	0.0216 (8)	0.0035 (6)	0.0007 (6)	−0.0043 (6)
C7	0.0227 (8)	0.0120 (7)	0.0233 (8)	0.0009 (6)	−0.0018 (7)	−0.0007 (6)
C8	0.0242 (9)	0.0237 (8)	0.0147 (8)	0.0015 (7)	−0.0005 (6)	0.0045 (6)
C9	0.0121 (8)	0.0301 (10)	0.0256 (9)	0.0001 (6)	−0.0009 (7)	0.0016 (7)
C10	0.0205 (8)	0.0180 (7)	0.0141 (7)	−0.0037 (6)	−0.0001 (6)	−0.0028 (6)
C11	0.0211 (8)	0.0176 (7)	0.0150 (7)	−0.0011 (6)	0.0032 (6)	−0.0043 (6)
C12	0.0225 (8)	0.0152 (7)	0.0219 (8)	−0.0039 (6)	0.0014 (7)	0.0021 (6)

### Geometric parameters (Å, °)

**Table:**

Ca1—O1	2.4046 (12)	C3—H3B	0.9900
Ca1—O2	2.3872 (12)	C4—H4A	0.9800
Ca1—O3	2.4871 (11)	C4—H4B	0.9800
Ca1—O4	2.4415 (11)	C4—H4C	0.9800
Ca1—O5	2.4254 (12)	C5—H5A	0.9800
Ca1—O6	2.5138 (11)	C5—H5B	0.9800
Ca1—I1	3.0525 (3)	C5—H5C	0.9800
O1—C2	1.4325 (19)	C6—C7	1.502 (2)
O1—C1	1.4421 (18)	C6—H6A	0.9900
O2—C3	1.437 (2)	C6—H6B	0.9900
O2—C4	1.439 (2)	C7—H7A	0.9900
O3—C6	1.4369 (19)	C7—H7B	0.9900
O3—C5	1.4489 (18)	C8—H8A	0.9800
O4—C7	1.4427 (19)	C8—H8B	0.9800
O4—C8	1.4490 (19)	C8—H8C	0.9800
O5—C10	1.4390 (19)	C9—H9A	0.9800
O5—C9	1.446 (2)	C9—H9B	0.9800
O6—C11	1.4425 (19)	C9—H9C	0.9800
O6—C12	1.4473 (19)	C10—C11	1.509 (2)
C1—H1A	0.9800	C10—H10A	0.9900
C1—H1B	0.9800	C10—H10B	0.9900
C1—H1C	0.9800	C11—H11A	0.9900
C2—C3	1.506 (2)	C11—H11B	0.9900
C2—H2A	0.9900	C12—H12A	0.9800
C2—H2B	0.9900	C12—H12B	0.9800
C3—H3A	0.9900	C12—H12C	0.9800
			
O2—Ca1—O1	68.51 (4)	O2—C4—H4B	109.5
O2—Ca1—O5	99.51 (4)	H4A—C4—H4B	109.5
O1—Ca1—O5	139.51 (4)	O2—C4—H4C	109.5
O2—Ca1—O4	87.03 (4)	H4A—C4—H4C	109.5
O1—Ca1—O4	136.26 (4)	H4B—C4—H4C	109.5
O5—Ca1—O4	78.05 (4)	O3—C5—H5A	109.5
O2—Ca1—O3	87.46 (4)	O3—C5—H5B	109.5
O1—Ca1—O3	75.70 (4)	H5A—C5—H5B	109.5
O5—Ca1—O3	144.21 (4)	O3—C5—H5C	109.5
O4—Ca1—O3	67.23 (4)	H5A—C5—H5C	109.5
O2—Ca1—O6	83.52 (4)	H5B—C5—H5C	109.5
O1—Ca1—O6	73.76 (4)	O3—C6—C7	107.98 (13)
O5—Ca1—O6	66.36 (4)	O3—C6—H6A	110.1
O4—Ca1—O6	140.91 (4)	C7—C6—H6A	110.1
O3—Ca1—O6	149.37 (4)	O3—C6—H6B	110.1
O2—Ca1—I1	166.17 (3)	C7—C6—H6B	110.1
O1—Ca1—I1	98.31 (3)	H6A—C6—H6B	108.4
O5—Ca1—I1	93.32 (3)	O4—C7—C6	108.59 (13)
O4—Ca1—I1	100.74 (3)	O4—C7—H7A	110.0
O3—Ca1—I1	85.10 (3)	C6—C7—H7A	110.0
O6—Ca1—I1	97.06 (3)	O4—C7—H7B	110.0
C2—O1—C1	111.03 (12)	C6—C7—H7B	110.0
C2—O1—Ca1	116.92 (9)	H7A—C7—H7B	108.4
C1—O1—Ca1	122.09 (9)	O4—C8—H8A	109.5
C3—O2—C4	112.11 (13)	O4—C8—H8B	109.5
C3—O2—Ca1	113.70 (9)	H8A—C8—H8B	109.5
C4—O2—Ca1	124.09 (10)	O4—C8—H8C	109.5
C6—O3—C5	111.16 (12)	H8A—C8—H8C	109.5
C6—O3—Ca1	108.91 (9)	H8B—C8—H8C	109.5
C5—O3—Ca1	120.68 (9)	O5—C9—H9A	109.5
C7—O4—C8	109.77 (12)	O5—C9—H9B	109.5
C7—O4—Ca1	117.68 (9)	H9A—C9—H9B	109.5
C8—O4—Ca1	129.75 (9)	O5—C9—H9C	109.5
C10—O5—C9	111.20 (12)	H9A—C9—H9C	109.5
C10—O5—Ca1	119.39 (9)	H9B—C9—H9C	109.5
C9—O5—Ca1	126.65 (10)	O5—C10—C11	107.67 (12)
C11—O6—C12	112.47 (12)	O5—C10—H10A	110.2
C11—O6—Ca1	102.93 (8)	C11—C10—H10A	110.2
C12—O6—Ca1	121.60 (9)	O5—C10—H10B	110.2
O1—C1—H1A	109.5	C11—C10—H10B	110.2
O1—C1—H1B	109.5	H10A—C10—H10B	108.5
H1A—C1—H1B	109.5	O6—C11—C10	111.17 (13)
O1—C1—H1C	109.5	O6—C11—Ca1	50.70 (7)
H1A—C1—H1C	109.5	C10—C11—Ca1	84.46 (9)
H1B—C1—H1C	109.5	O6—C11—H11A	109.4
O1—C2—C3	108.45 (13)	C10—C11—H11A	109.4
O1—C2—H2A	110.0	Ca1—C11—H11A	159.8
C3—C2—H2A	110.0	O6—C11—H11B	109.4
O1—C2—H2B	110.0	C10—C11—H11B	109.4
C3—C2—H2B	110.0	Ca1—C11—H11B	79.7
H2A—C2—H2B	108.4	H11A—C11—H11B	108.0
O2—C3—C2	108.03 (13)	O6—C12—H12A	109.5
O2—C3—H3A	110.1	O6—C12—H12B	109.5
C2—C3—H3A	110.1	H12A—C12—H12B	109.5
O2—C3—H3B	110.1	O6—C12—H12C	109.5
C2—C3—H3B	110.1	H12A—C12—H12C	109.5
H3A—C3—H3B	108.4	H12B—C12—H12C	109.5
O2—C4—H4A	109.5		
